# ﻿Two new species of *Xylaria* (Xylariaceae, Ascomycota) associated with fallen leaves in Hainan Tropical Rainforest National Park, China

**DOI:** 10.3897/mycokeys.112.140487

**Published:** 2025-01-07

**Authors:** Xiaoyan Pan, Zongzhu Chen, Yiqing Chen, Jinrui Lei, Xiaohua Chen, Tingtian Wu, Yuanling Li

**Affiliations:** 1 Hainan Academy of Forestry (Hainan Academy of Mangrove), Haikou, 571100, China Hainan Academy of Forestry (Hainan Academy of Mangrove) Haikou China; 2 Key Laboratory of Tropical Forestry Resources Monitoring and Application of Hainan Province, Haikou, 571100, China Key Laboratory of Tropical Forestry Resources Monitoring and Application of Hainan Province Haikou China

**Keywords:** Foliicolous, molecular phylogenetics, morphology, *Xylaria*, taxonomy

## Abstract

*Xylaria* is a large and complex genus of macrofungi, playing a critical role in ecosystems as decomposers and possessing antimicrobial, anticancer, and antioxidant properties. This paper described two new *Xylaria* species discovered on fallen leaves in Hainan Tropical Rainforest National Park, based on morphological characteristics and genetic sequences. Detailed color illustrations and comparisons with similar species were provided. The main characteristics of *X.acifer* included long, needle-shaped stromata, ellipsoid to fusoid ascospores, and prominent non-cellular appendages. The stromata of *X.tuberculosa* had an aggregated, knobby fertile part, with smaller ascospores also featuring non-cellular appendages. Additionally, a key to 46 *Xylaria* species associated with fallen leaves and petioles worldwide was established.

## ﻿Introduction

*Xylaria* Hill ex Schrank, established in 1789, is the type genus of the Xylariaceae family, with *X.hypoxylon* (L.) Grev. as the type species. Most *Xylaria* species are saprotrophic, a few are parasitic, and some form symbiotic relationships with plants as endophytes (Martín 1970; Rogers 1979; Deshmukh et al. 2022). These fungi are widely distributed across tropical, subtropical, and temperate regions (Ma et al. 2022) and are commonly referred to as “dead man’s fingers” (Deshmukh et al. 2022). *Xylaria* species exhibit unique survival adaptations, and their melanin production creates a physical and chemical barrier that other competing fungi cannot penetrate, allowing them to expel excess water in extremely humid environments and thrive in harsh conditions (Rajtar et al. 2023).

To date, *Xylaria* species have been reported on six of the seven continents (Dennis 1956, 1958; Greenhalgh and Chesters 1968; Rogers and Samuels 1986; Whalley 1996; Ju and Rogers 1999; Rogers 2000; San Martín et al. 2001). Most known *Xylaria* species grow on wood, while a minority are found on fallen leaves and petioles, fallen fruits and seeds, and termite nests and soil (Rogers et al. 2005; Ju and Hsieh 2007; Ju et al. 2018; Fournier et al. 2020; Wangsawat et al. 2021; Hsieh et al. 2022; Ju et al. 2022; Ju and Hsieh 2023). Fallen leaves and petioles are important substrates for *Xylaria* species, but these species are often difficult to detect due to their fragile, small, and sparse stromata (Ju and Hsieh 2023; Pan et al. 2024). It is also common for different species stromata to grow on the same leaf, complicating the study of *Xylaria* species associated with these substrates (Ju and Hsieh 2023; Pan et al. 2024).

Another challenge in identifying *Xylaria* species associated with fallen leaves and petioles arises from *X.filiformis* (Alb. & Schwein.) Fr. Studies on *Xylaria* species related to fallen leaves and petioles worldwide often reference *X.filiformis* (Ju and Hsieh 2023), but the identification of this species is rather problematic. *Xylariafiliformis*, originally described as *Sphaeriafiliformis* Alb. & Schwein. in 1805, has subsequently been classified as *Hypoxylonfiliforme* (Alb. & Schwein.) Rabenh., *Xylosphaerafiliformis* (Alb. & Schwein.) Dennis, and *Podosordariafiliformis* (Alb. & Schwein.) P.M.D. Martin (http://www.indexfungorum.org/, 29 October 2024). Ju and Hsieh (2023) clarified the identity of *X.filiformis*, systematically categorizing 42 species associated with fallen leaves and petioles and making significant contributions to the taxonomy of this group. Pan et al. (2024) further expanded this group by describing *X.diaoluoshanensis* Xiao Y. Pan and *X.fulvotomentosa* Xiao Y. Pan, increasing the number of species to 44. In this paper, we continued to explore this substrate group by describing two new species, *X.acifer* and *X.tuberculosa*, based on morphological characteristics and molecular sequences. Additionally, we provide a key to 46 *Xylaria* species associated with fallen leaves and petioles.

## ﻿Materials and methods

### ﻿Specimen source and morphological study

The specimens were collected from the Diaoluoshan Area of Hainan Tropical Rainforest National Park and are currently stored at the Forest Resources Institute of Hainan Academy of Forestry. Habitat photographs of the specimens were taken using a Canon D3 camera (Canon Corporation, Tokyo, Japan) and a Huawei Mate 50 smartphone (Huawei Technologies Co., Ltd., Shenzhen, China). The macroscopic characteristics of the specimens were examined using a VHX-5000 digital microscope (Keyence Corporation, Osaka, Japan). Microscopic morphological features were observed and measured with a fully automatic optical microscope DM6B (Leica Corporation, Wetzlar, Germany). In this study, N represented the number of ascospores observed and measured, and M represented the average size of the ascospores.

### ﻿DNA extraction and amplification

Total DNA from the specimens was extracted using the cetyltrimethylammonium bromide (CTAB) Plant Genome Rapid Extraction Kit (Aidlab Biotechnologies, Beijing, China). This DNA was used to amplify gene sequences from three loci: ITS, RPB2, and TUB2. Each PCR reaction was performed in a 40 µL system (16 µL ddH_2_O, 20 µL HS™ Mix, 1 µL forward primer, 1 µL reverse primer, and 2 µL DNA template). The ITS region was amplified using primers ITS4/ITS5 (White et al. 1990) with the following PCR program: initial denaturation at 95 °C for 3 minutes, followed by 30 cycles of 94 °C for 40 seconds, 55.8 °C for 45 seconds, and 72 °C for 1 minute, with a final extension at 72 °C for 10 minutes (Pan et al. 2022). RPB2 was amplified using primers 7CR/5F (Liu et al. 1999) and RPB2AM-1bf/RPB2AM-7R (Miller and Huhndorf 2005), and TUB2 was amplified using primers T1/T22 (O’Donnell and Cigelnik 1997). The PCR program for both RPB2 and TUB2 was: initial denaturation at 95 °C for 3 minutes, followed by 35 cycles of 94 °C for 1 minute, 52 °C for 1 minute, and 72 °C for 1.5 minutes, with a final extension at 72 °C for 10 minutes (Hsieh et al. 2005). The PCR products were sequenced by Tianyi Huiyuan Gene Technology Co., Ltd. (Wuhan, China). The obtained sequences were submitted to GenBank to receive accession numbers.

### ﻿Phylogenetic analysis

In this study, ten newly extracted sequences were analyzed alongside 256 Xylariaceae sequences collected from the National Center for Biotechnology Information (NCBI), resulting in a total of 266 gene sequences (Table 1). These included 100 ITS sequences, 85 TUB2 sequences, and 81 RPB2 sequences. A phylogenetic tree was constructed based on the ITS-TUB2-RPB2 dataset. Sequences were verified and aligned using the online tool MAFFT v.7 (http://mafft.cbrc.jp/alignment/server/), manually trimmed and optimized in BioEdit v. 7.0.5.2 (Hall 1999), and the three loci sequences were concatenated using MEGA v.6.0 (Felsenstein 1981; Tamura et al. 2011). Phylogenetic analysis was conducted using both maximum likelihood (ML) and Bayesian inference (BI) methods. ML analysis was performed in RAxML v.8.2.10 (Stamatakis 2014), while BI analysis was conducted using MrBayes v.3.2.6 (Huelsenbeck and Ronquist 2001; Ronquist et al. 2012; Song et al. 2022). *Poroniapileiformis* (Berk.) Fr. was selected as the outgroup. The phylogenetic tree was viewed and adjusted in FigTree v.1.4.3.

**Table 1.** List of species, specimens, and gene sequence accession numbers used in this study. New sequences provided in this study are in bold. “NA”= not available.

Taxon	Substrate/Origin	Specimen No.	GenBank No.			Reference
			ITS	TUB2	RPB2	
*Poroniapileiformis*	Cow dung/China	WSP 88113001	GU324760	GQ502720	GQ853037	Hsieh et al. (2010)
***Xylariaacifer***	**Fallen leaves/China**	**HAFFR 122**	**PQ483147**	**PQ498329**	**PQ498326**	**This study**
***X.acifer***	**Fallen leaves/China**	**HAFFR 130**	**PQ483150**	NA	**PQ498328**	**This study**
*X.acuminatilongissim*a	Termite nests/China	HAST 623	EU178738	GQ502711	GQ853028	Hsieh et al. (2010)
*X.aethiopica*	Pods of *Millettiaferrugine*a/Ethiopia	YMJ 1136	MH790445	MH785221	MH785222	Fournier et al. (2018)
*X.adscenden*s	Wood/Guadeloupe	HAST 570	GU300101	GQ487708	GQ844817	Hsieh et al. (2010)
*X.allantoidea*	Trunk/China	HAST 94042903	GU324743	GQ502692	GQ848356	Hsieh et al. (2010)
*X.amphithele*	Dead leaves/Guadeloupe	HAST 529	GU300083	GQ478218	GQ844796	Hsieh et al. (2010)
*X.apoda*	Bark/China	HAST 90080804	GU322437	GQ495930	GQ844823	Hsieh et al. (2010)
*X.arbuscula*	Bark/China	HAST 89041211	GU300090	GQ478226	GQ844805	Hsieh et al. (2010)
X.arbusculavar.plenofissura	Wood/China	HAST 93082814	GU339495	GQ478225	GQ844804	Hsieh et al. (2010)
*X.aristata*	Petioles/Indonesia	YMJ 1823	OQ883719	NA	NA	Ju and Hsieh (2023)
*X.atrodivaricat*a	Termite nests/China	HAST 95052001	EU178739	GQ502713	GQ853030	Hsieh et al. (2010)
*X.badia*	Bamboo culm/China	HAST 95070101	GU322446	GQ495939	GQ844833	Hsieh et al. (2010)
*X.bambusicola*	Bamboo culm/Thailand	JDR 162	GU300088	GQ478223	GQ844801	Hsieh et al. (2010)
*X.berteri*	Bark/USA	JDR 256	GU324750	GQ502698	GQ848363	Hsieh et al. (2010)
*X.berteri*	Bark/China	HAST 90112623	GU324749	AY951763	GQ848362	Hsieh et al. (2010)
*X.betulicola*	Leaves of *Betula*/China	FCATAS 750	MF774332	NA	NA	Ma and Li (2018)
*X.brevifurcat*a	Ground/China	YMJ 100062601	OQ845482	OQ818432	OQ851526	Ju et al. (2023)
*X.brunneovinos*a	Termite nests/China	HAST 720	EU179862	GQ502706	GQ853023	Hsieh et al. (2010)
*X.castorea*	Wood/New Zealand	PDD 600	GU324751	GQ502703	GQ853018	Hsieh et al. (2010)
*X.chaiyaphumensi*s	Termite nests/Thailand	SWUF16-11.4	MT622776	OQ845433	OQ851585	Wangsawat et al. (2021); Ju et al. (2023)
*X.cirrat*a	Termite nests/China	HAST 664	EU179863	GQ502707	GQ853024	Hsieh et al. (2010)
*X.coccophora*	Wood/French	HAST 786	GU300093	GQ487701	GQ844809	Hsieh et al. (2010)
*X.crinalis*	Wood/China	FCATAS 751	MF774330	NA	NA	Ma and Li (2018)
*X.crozonensis*	Bark/France	HAST 398	GU324748	GQ502697	GQ848361	Hsieh et al. (2010)
*X.cubensis*	Log/Russian Far East	HAST 477	NA	GQ502699	GQ848364	Hsieh et al. (2010)
*X.culleniae*	Pod/Thailand	JDR 189	GU322442	GQ495935	GQ844829	Hsieh et al. (2010)
*X.delicatula*	On decaying leaves/French Guiana	GS 2775	OQ883720	NA	NA	Ju and Hsieh (2023)
*X.diaoluoshanensis*	Fallen leaves/China	HAFFR 115	OR702611	OR726655	NA	Pan et al. (2024)
*X.diaoluoshanensis*	Fallen leaves/China	HAFFR 117	OR702612	OR726656	OR757125	Pan et al. (2024)
*X.diaoluoshanensis*	Fallen leaves/China	HAFFR 127	OR702613	OR726657	NA	Pan et al. (2024)
*X.fabacearum*	Seed pods of Fabaceae/Thailand	MFLU 16-1061	NR171104	MT212220	MT212202	Perera et al. (2020)
*X.fabaceicola*	Seed pods of Fabaceae/Thailand	MFLU 16-1072	NR171103	MT212219	MT212201	Perera et al. (2020)
*X.feejeensi*s	Bark/China	HAST 92092013	GU322454	GQ495947	GQ848336	Hsieh et al. (2010)
*X.ficicol*a	Fallen leaves and petioles of *Ficusauriculata*/China	HMJAU 22818	MZ351258	NA	NA	Pan et al. (2022)
*X.filiformis*	Herbaceous stem/Iran	GUM 1052	KP218907	NA	NA	Hashemi et al. (2015)
*X.fimbriat*a	Termite nests/French West Indies	HAST 491	GU324753	GQ502705	GQ853022	Hsieh et al. (2010)
*X.fulvotomentosa*	Fallen leaves/China	HAFFR 124	OR702619	OR726658	OR757121	Pan et al. (2024)
*X.fulvotomentosa*	Fallen leaves/China	HAFFR 129	OR702620	OR726659	OR757122	Pan et al. (2024)
*X.furcata*	Termite nests/China	HAST 646	GU324757	GQ502715	GQ853032	Hsieh et al. (2010)
*X.furcatula*	Ground/China	YMJ 98070105	OQ845483	OQ818433	OQ851527	Ju et al. (2023)
X.cf.glebulosa	Fruit/French West Indies	HAST 431	GU322462	GQ495956	GQ848345	Hsieh et al. (2010)
*X.grammica*	Wood/China	HAST 479	GU300097	GQ487704	GQ844813	Hsieh et al. (2010)
*X.griseosepiace*a	Termite nests/China	HAST 641	EU179865	GQ502714	GQ853031	Hsieh et al. (2010)
*X.hedyosmicola*	Fallen leaves of *Hedyosmumorientale*/China	FCATAS 856	MZ227121	MZ221183	MZ683407	Pan et al. (2022)
*X.hedyosmicola*	Fallen leaves of *Hedyosmumorientale*/China	FCATAS 857	MZ227023	MZ221184	MZ851780	Pan et al. (2022)
*X.hoehneli*i	Ground/China	YMJ 95052006	OQ845489	OQ818439	OQ851533	Ju et al. (2023)
*X.hypoxylon*	Wood/Belgium	HAST 152	GU300096	GQ260187	GQ844812	Hsieh et al. (2010)
*X.hypoxylon*	Wood/China	HAST 95082001	GU300095	GQ487703	GQ844811	Hsieh et al. (2010)
*X.ianthinovelutina*	Fruit of *Swietenia*/Martinique	HAST 553	GU322441	GQ495934	GQ844828	Hsieh et al. (2010)
*X.insignifurcata*	Ground/China	YMJ 95071201	OQ845496	OQ818446	OQ851540	Ju et al. (2023)
*X.intraflav*a	Termite nests/China	HAST 725	EU179866	GQ502718	GQ853035	Hsieh et al. (2010)
*X.juruensis*	*Arengaengleri*/China	HAST 92042501	GU322439	GQ495932	GQ844825	Hsieh et al. (2010)
*X.laevis*	Wood/Martinique	HAST 419	GU324746	GQ502695	GQ848359	Hsieh et al. (2010)
*X.leavis*	Bark/China	HAST 95072910	GU324747	GQ502696	GQ848360	Hsieh et al. (2010)
*X.lindericola*	Fallen leaves of *Linderarobusta*/China	FCATAS 852	MZ005635	MZ031978	MZ031982	Pan et al. (2022)
*X.lindericola*	Fallen leaves of *Linderarobusta*/China	FCATAS 853	MZ005636	MZ031979	MZ048749	Pan et al. (2022)
*X.liquidambaris*	Fruits of *Liquidambarformosana*/China	HAST 93090701	GU300094	GQ487702	GQ844810	Hsieh et al. (2010)
*X.longissima*	Wood/China	FCATAS 749	MF774331	NA	NA	Ma and Li (2018)
*X.longissima*	Wood/Iran	IRAN 16582 F	KP218906	NA	NA	Hashemi et al. (2015)
*X.meliacearum*	Petioles and infructescence of *Guareaguidonia*/Puerto Rico	JDR 148	GU300084	GQ478219	GQ844797	Hsieh et al. (2010)
*X.minuscula*	Fallen leaves of *Castanopsiscarlesiivar.Sessilis*/China	YMJ 90102701	OQ883721	NA	NA	Ju and Hsieh (2023)
*X.multiplex*	Wood/USA	JDR 259	GU300099	GQ487706	GQ844815	Hsieh et al. (2010)
*X.muscula*	Dead branch/French West	HAST 520	GU300087	GQ478222	GQ844800	Hsieh et al. (2010)
*X.nigripe*s	Termite nests/China	HAST 653	GU324755	GQ502710	GQ853027	Hsieh et al. (2010)
*X.oxyacanthae*	Fallen seeds/USA	JDR 859	GU322434	GQ495927	GQ844820	Hsieh et al. (2010)
*X.oxyacanthae*	Fruits/Germany	LZ 2010-502	HQ414587	NA	NA	Roensch et al. (2010)
*X.palmicola*	Fruits/New Zealand	PDD 604	GU322436	GQ495929	GQ844822	Hsieh et al. (2010)
*X.petchii*	Fallen branches/China	HAFFR 60	OR702616	OR735171	NA	Pan et al. (2024)
*X.petchii*	Fallen leaves of *Daphniphyllumpaxianum*/China	HAFFR 118	OR702617	OR735172	OR757123	Pan et al. (2024)
*X.petchii*	Fallen leaves of *Daphniphyllumpaxianum*/China	HAFFR 126	OR702618	OR735173	OR757124	Pan et al. (2024)
*X.phyllocharis*	Dead leaves/French West	HAST 528	GU322445	GQ495938	GQ844832	Hsieh et al. (2010)
*X.plebej*a	Trunk/China	HAST 91122401	GU324740	GQ502689	GQ848353	Hsieh et al. (2010)
*X.polymorpha*	Wood/USA	JDR 1012	GU322460	GQ495954	GQ848343	Hsieh et al. (2010)
*X.polymorpha*	Stump/Germany	M:M-0125909	FM164944	NA	NA	Persoh et al. (2009)
*X.polysporicola*	Fallen leaves of *Polysporahainanensis*/China	FCATAS 848	MZ005592	MZ031976	MZ031980	Pan et al. (2022)
*X.polysporicola*	Fallen leaves of *Polysporahainanensis*/China	FCATAS 849	MZ005591	MZ031977	MZ031981	Pan et al. (2022)
*X.reevesia*e	Fruits of *Reevesiaformosana*/China	HAST 90071609	GU322435	GQ495928	GQ844821	Hsieh et al. (2010)
*X.regalis*	Log of *Ficusracemose*/India	HAST 920	GU324745	GQ502694	GQ848358	Hsieh et al. (2010)
*X.rogersi*i	Fruits of *Magnolia* sp./China	FCATAS 915	MZ648827	NA	MZ707121	Ma et al. (2022)
*X.schimicol*a	Fruits of *Schimanoronhae*/China	FCATAS 896	MZ648850	MZ695787	MZ707114	Ma et al. (2022)
*X.schweinitzii*	Bark/China	HAST 92092023	GU322463	GQ495957	GQ848346	Hsieh et al. (2010)
*X.scopar*ia	Ground/China	YMJ 97072802	OQ843005	OQ818480	OQ851574	Ju et al. (2023)
*X.siamensis*	Termite nests/Thailand	SWUF 17-20.2	MT622765	OQ845437	OQ851589	Wangsawat et al. (2021); Ju et al. (2023)
X.siculaf.major	Fallen leaves/China	HAST 90071613	GU300081	GQ478216	GQ844794	Hsieh et al. (2010)
*X.sihanonthii*	Termite nests/Thailand	SWUF 18-1.3	MT622785	MW459242	NA	Wangsawat et al. (2021)
*X.simplicissima*	On herbaceous stems/Finland	MP 111004	OQ883722	NA	NA	Ju and Hsieh (2023)
*X.striata*	Branch/China	HAST 304	GU300089	GQ478224	GQ844803	Hsieh et al. (2010)
*X.subintraflava*	Termite nests/Thailand	SWUF 16-4.3	MT622762	OQ845438	OQ851590	Wangsawat et al. (2021); Ju et al. (2023)
*X.tenellifurcata*	Ground/China	YMJ 98052001	OQ845498	OQ818448	OQ851542	Ju et al. (2023)
*X.theaceicol*a	Fruits of *Schimavillosa*/China	FCATAS 903	MZ648848	MZ695788	MZ707115	Ma et al. (2022)
*X.thienhirunae*	Termite nests/Thailand	SWUF 16-6.2	MT622770	MW459235	NA	Wangsawat et al. (2021)
***X.tuberculosa***	**Fallen leaves/China**	**HAFFR 63**	**PQ483149**	**PQ498331**	NA	**This stud**y
***X.tuberculosа***	**Fallen leaves/China**	**HAFFR 123**	**PQ483148**	**PQ498330**	**PQ498327**	**This study**
*X.venosula*	Twigs/USA	HAST 94080508	EF026149	EF025617	GQ844806	Hsieh et al. (2010)
*X.venustula*	Bark/China	HAST 88113002	GU300091	GQ487699	GQ844807	Hsieh et al. (2010)
*X.vittatipiliformis*	Dead leave/Guadeloupe	CLLGUAD 029	OQ883723	NA	NA	Ju and Hsieh (2023)
*X.vivanti*i	Fruits of *Magnolia* sp./Martinique	HAST 519	GU322438	GQ495931	GQ844824	Hsieh et al. (2010)
*X.wallichii*	Fruits of *Schimawallichii*/China	FCATAS 923	MZ648861	MZ695793	MZ707118	Ma et al. (2022)
*X.xylarioides*	Wood/Iran	GUM 1051	KP218909	NA	NA	Hashemi et al. (2015)

## ﻿Results

### ﻿Molecular phylogeny

**Figure 1.**
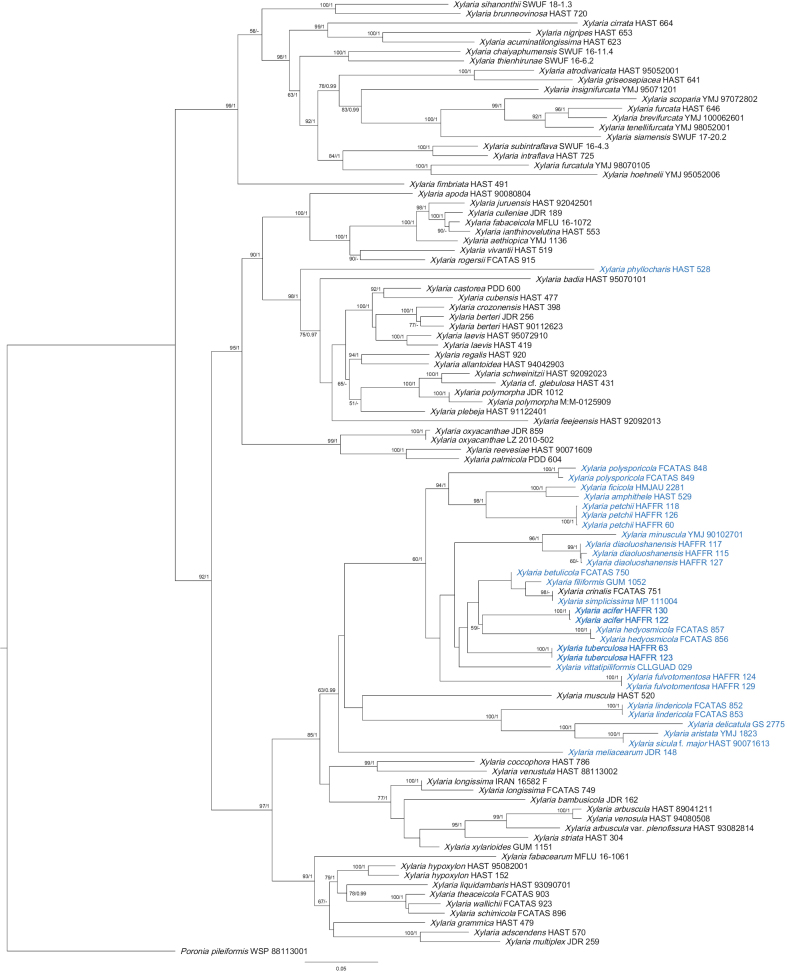
Phylogenetic tree of *Xylaria* based on the ITS-TUB2-RPB2 sequence dataset constructed using ML analysis. Support values from ML and BI analyses (bootstrap supports ≥ 50%, posterior probability values ≥ 0.95) are indicated above or below the respective branches (ML/BI). Species associated with fallen leaves and petioles are marked in blue, and the species described in this paper are in bold.

After alignment in MAFFT v.7, the character positions for the ITS, TUB2, and RPB2 sequences were determined to be 822, 2112, and 1255, respectively. After post-trimming, the ITS sequence retained 520 character positions, and TUB2 and RPB2 retained 1525 and 1027, respectively. The concatenated ITS-TUB2-RPB2 sequence dataset thus consisted of 3072 characters, including 1374 parsimony-informative positions. The results of the phylogenetic analyses showed no significant differences between the BI and ML trees. Bayesian posterior probabilities (≥ 0.95) and RAxML bootstrap values (≥ 50%) were marked on the phylogenetic tree (Fig. 1). In the phylogenetic tree, *X.acifer* clustered together with *X.hedyosmicola*, Hai X. Ma & X.Y. Pan, while *X.tuberculosa* was closely related to *X.acifer*, *X.betulicola*, Hai X. Ma, Lar.N. Vassiljeva & Yu Li, *X.crinalis*, Hai X. Ma, Lar.N. Vassiljeva & Yu Li, *X.filiformis*, *X.hedyosmicola*, and *X.simplicissima* (Pers.) Y.M. Ju & H.M. Hsieh.

### ﻿Taxonomy

#### ﻿ Xylaria acifer

Taxon classificationFungiXylarialesXylariaceae

Xiao Y. Pan sp. nov.

F10FD265-F353-5914-B4D8-FA1672AF05B2

MycoBank No: 856020

Fig. 2

##### Holotype.

China • Hainan Province, Diaoluoshan Area of Hainan Tropical Rainforest National Park; 18°43'35"N, 109°52'11"E; elevation 973 m; on fallen leaves, 18 June 2023, Xiaoyan Pan (HAFFR 122). GenBank accession numbers PQ483147 (ITS), PQ498329 (TUB2), and PQ498326 (RPB2).

**Figure 2.**
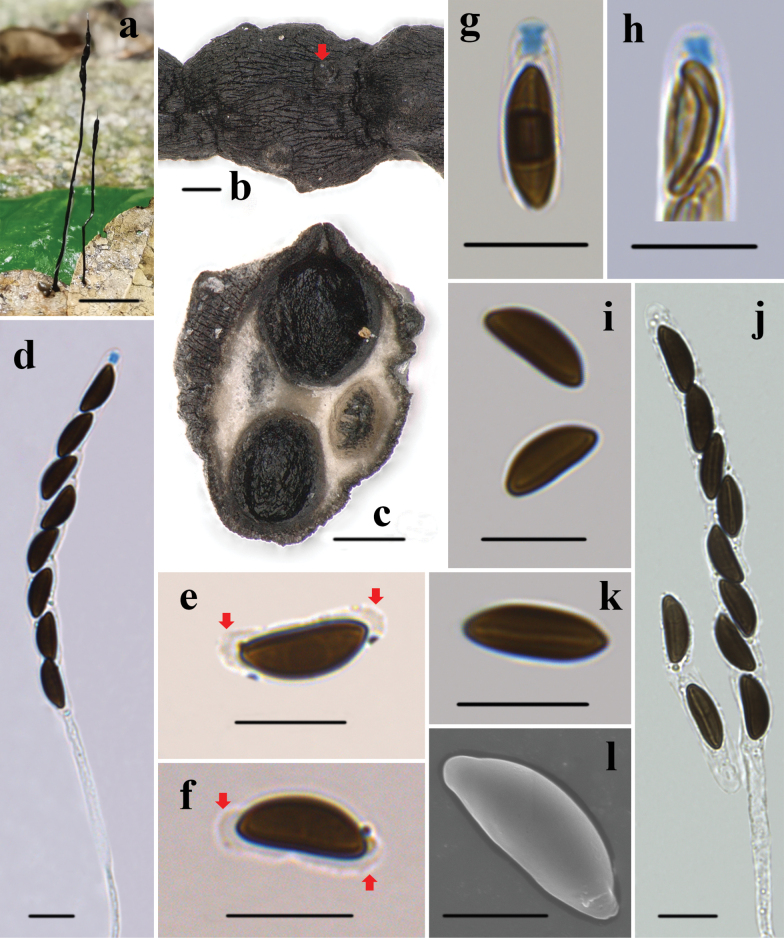
*Xylariaacifer* (HAFFR 122) a stromata on leaves **b** stromatal surface and ostioles (arrow) **c** section through stroma, showing perithecia **d** ascus in Melzer’s reagent **e, f** ascospore showing non-cellular appendages in 1% SDS (arrows) **g, h** ascal apical ring in Melzer’s reagent **i** ascospores in Melzer’s reagent **j** ascus in water **k** ascospore showing a nearly spore-length straight germ slit in Melzer’s reagent **l** ascospore under SEM. Scale bars: 1 cm (**a**); 200 µm (**b, c**); 10 µm (**d–k**); 5 µm (**l**).

##### Diagnosis.

Differs from *X.hedyosmicola* by its smaller ascospores. Differs from *X.vittatipiliformis* by the absence of band-like stripes on its stromata surfaces and its smaller ascospores. Differs from *X.vittiformis* by the absence of band-like stripes on its stromata surfaces.

##### Etymology.

“*acifer*” refers to the needle-like shape of the stromata.

##### Teleomorph.

***Stromata*** upright, solitary, needle-like, unbranched, 25–50 mm total length; acute sterile apex, 2–10 mm; fertile portion 3–10 mm long × 0.8–1.2 mm diam., elongate cylindrical, composed of tightly arranged perithecia; stipe glabrous, 15–40 mm long × 0.5–1 mm diam., longitudinally striate, the base slightly swollen; surface roughened, black, with conspicuous to half-exposed perithecial mounds; interior white to creamy; texture soft. ***Perithecia*** spherical, 250–500 µm diam. ***Ostioles*** papillate. ***Asci*** with eight ascospores arranged in uniseriate manner, cylindrical, 90–150 µm total length, spore-bearing part 60–85 µm long × 5.5–7.5 µm wide, stipe 30–70 µm long, with a bluing apical ring in Melzer’s reagent, tubular to slightly urn-shaped, 1.8–3.5 µm high × 1.4–2.5 µm diam. ***Ascospores*** brown to dark brown, unicellular, ellipsoid to fusiform, inequilateral, with narrowly rounded ends, smooth, (9.3–)10–11(–12) × 3.7–4.7 µm (M = 10.6 × 4.2 µm, N = 40), with straight germ slit nearly the full length of the spore on the flattened side, a hyaline sheath visible in 1% SDS, swollen at both ends, forming non-cellular appendages.

##### Remarks.

*Xylariaacifer* clusters with *X.hedyosmicola* in the phylogenetic tree, and they share some similarities in stromatal morphology, yet they also exhibit distinct differences. The stromata of *X.hedyosmicola* possess more prominent perithecial mounds (half-exposed to fully exposed), and its ascospores are brown, ellipsoid, and larger ((12–)13–15(–16.7) × (6–) 6.5–7.5 (–8.5) µm) (Pan et al. 2022). *Xylariavittatipiliformis* Y.-M. Ju, H.-M. Hsieh & Fournier and *X.vittiformis* Y.-M. Ju & H.-M. Hsieh are morphologically similar to *X.acifer*. *Xylariavittatipiliformis* can be distinguished by its band-like striped grayish brown outer peeling layer and larger ascospores ((10–)11–12(–12.5) × (5.5–)6–7(–7.5) µm) (Ju and Hsieh 2023). *Xylariavittiformis* is separated from *X.acifer* by its grayish brown outer peeling layer split into band-like stripes, smaller perithecia (150–200 μm broad), and ellipsoid ascospores, (8–)8.5–10(–11) × (4–)4.5–5(–5.5) µm (Ju and Hsieh 2023).

*Xylariaappendiculatoides* Y.M. Ju & H.M. Hsieh, *X.filiformis*, and *X.simplicissima* are also similar to *X.acifer* in stromatal morphology but are distinctly different. *Xylariaappendiculatoides* has stromata with sharper ostioles (conic-papillate, tilting upwards) and larger ascospores ((14–)15–16(–17) × (6.5–)7.0–7.5(–8) µm) (Ju and Hsieh 2023). *Xylariafiliformis* and *X.simplicissima* have ascospores that are markedly different from those of *X.acifer*. *Xylariafiliformis* has light brown ascospores that are larger ((9.5–)11.5–13.5(–14.5) × (4–)4.5–5.5(–6) µm) (Ju and Hsieh 2023). *Xylariasimplicissima* has larger ascospores ((15–)16.5–19(–21.5) × (5–)5.5–6.5(–7.5) µm) that lack non-cellular appendages (Ju and Hsieh 2023). Additionally, *X.acifer*, *X.filiformis*, and *X.simplicissima* are distributed in different clades in the phylogenetic tree. *Xylariamaitlandii* (Dennis) D. Hawksw. has a somewhat similar stromatal shape to *X.acifer*. However, *X.maitlandii* has hairy stromata, which clearly distinguishes it from *X.acifer* (Ju and Hsieh 2023).

##### Additional specimen examined.

China • Hainan Province, Diaoluoshan Area of Hainan Tropical Rainforest National Park; 18°43'31"N, 109°52'14"E; elevation 937 m; on fallen leaves, 18 June 2023, Xiaoyan Pan (HAFFR 130). GenBank accession numbers PQ483150 (ITS) and PQ498328 (RPB2).

#### ﻿ Xylaria tuberculosa

Taxon classificationFungiXylarialesXylariaceae

Xiao Y. Pan sp. nov.

FF6C9A8B-E57D-5D7E-9E2A-5620AE7E46C8

MycoBank No: 856021

Fig. 3

##### Holotype.

China • Hainan Province, Diaoluoshan Area of Hainan Tropical Rainforest National Park; 18°43'33"N, 109°52'20"E; elevation 932 m; on fallen leaves, 18 June 2023, Xiaoyan Pan (HAFFR 123). GenBank accession numbers PQ483148 (ITS), PQ498330 (TUB2), and PQ498327 (RPB2).

**Figure 3.**
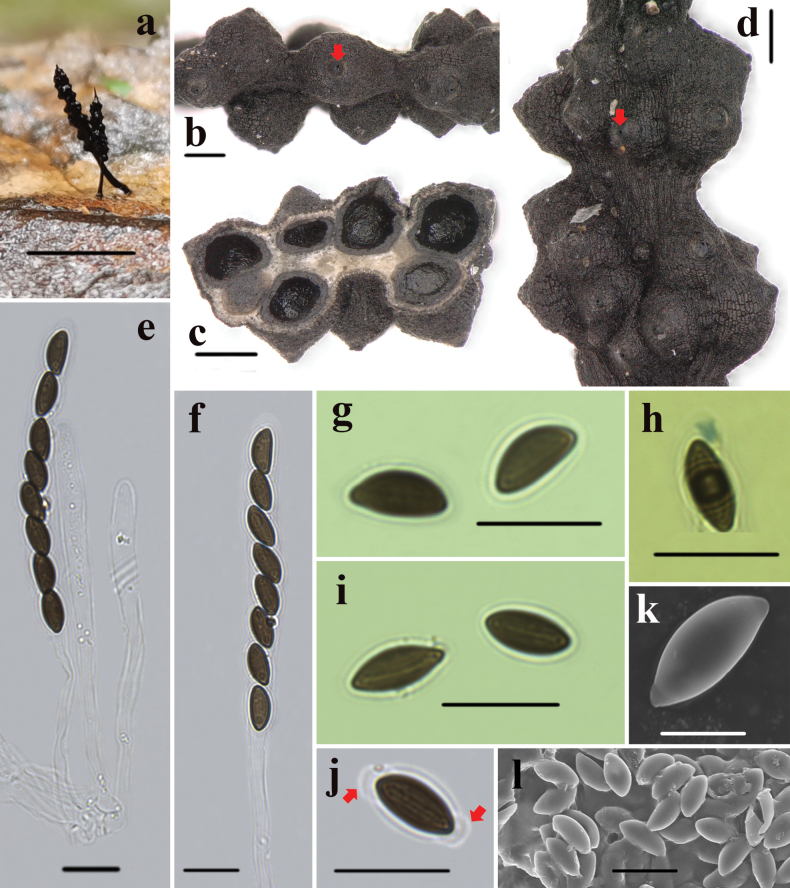
*Xylariatuberculosa* (HAFFR 123) a stromata on leaves **b, d** stromatal surface and ostioles (arrow) **c** section through stroma, showing perithecia **e, f** ascus in 1% SDS **g** ascospores in water **h** ascal apical ring in Melzer’s reagent **i** ascospore showing a slightly shorter than spore-length straight germ slit in water **j** ascospore showing non-cellular appendages in 1% SDS (arrows) **k, l** ascospores under SEM. Scale bars: 0.5 cm (**a**); 200 µm (**b–d**); 10 µm (**e–j, l**); 5 µm (**K**).

##### Diagnosis.

Differs from *X.acifer*, *X.hedyosmicola*, *X.petchii*, and *X.polysporicola* by having smaller ascospores.

##### Etymology.

“*tuberculosa*” refers to the appearance of the stromata surface, which resembles segmented tubercles formed by the clustered perithecia.

##### Teleomorph.

***Stromata*** upright, solitary to scattered, cylindrical, unbranched, 5–10 mm total length; acute sterile apex, 0.1 mm; fertile portion 3–6 mm long × 0.3–1 mm wide, cylindrical, composed of tightly packed perithecia; stipe glabrous, 1.8–5 mm long × 0.2–0.5 mm wide, longitudinally striate, the base slightly swollen; surface roughened, black, with half-exposed to fully exposed perithecial mounds; interior white to creamy; texture soft. ***Perithecia*** spherical, 180–350 µm diam. ***Ostioles*** papillate. ***Asci*** with eight ascospores arranged in uniseriate manner, cylindrical, 70–105 µm total length, spore-bearing part 50–70 µm long × 5–7 µm wide, stipe 25–45 µm long, with a bluing apical ring in Melzer’s reagent, tubular to slightly urn-shaped, 1.8–2.6 µm high × 1.2–2.5 µm wide. ***Ascospores*** brown, unicellular, ellipsoid, inequilateral, with narrowly rounded ends, smooth, 7–8(–9) × 3.3–4.3 µm (M = 7.6 × 3.8 µm, N = 40), with straight germ slit slightly shorter than the spore length on the flattened side, and a hyaline sheath visible in 1% SDS, swollen at both ends, forming non-cellular appendages.

##### Remarks.

*Xylariatuberculosa* forms a distinct branch in the phylogenetic tree with *X.acifer*, *X.betulicola*, *X.crinalis*, *X.filiformis*, *X.hedyosmicola*, *and X.simplicissima*, but it is morphologically different from these six species. *Xylariaacifer* has a needle-like stroma with flatter perithecial mounds and larger ascospores (9.3–12 × 3.7–4.7 µm), while *X.betulicola*, *X.crinalis*, *X.filiformis*, *X.hedyosmicola*, *and X.simplicissima* all have stromata with long sterile apices and significantly larger ascospores, measuring (9.5–21.5 × 3.5–8.5 µm). The ascospores of *X.betulicola*, *X.crinalis*, *X.filiformis*, *X.hedyosmicola*, and *X.simplicissima* measure (11.5–)12–14(–15) × 5–6 µm, (14–)15–16.5(–17.5) × (3.5–)4–5(–6) µm, (9.5–)11.5–13.5(–14.5) × (4–)4.5–5.5(–6) µm, (12–)13–15(–16.7) × (6–) 6.5–7.5 (–8.5) µm, and (15–)16.5–19(–21.5) × (5–)5.5–6.5(–7.5) µm, respectively (Ma and Li 2018; Pan et al. 2022; Ju and Hsieh 2023).

*Xylariapetchii* Lloyd and *X.polysporicola* Hai X. Ma & X.Y. Pan are similar to *X.tuberculosa* morphologically, as they share cylindrical stromata growing on fallen leaves. Pan et al. (2024) described *X.petchii* from China, noting that this species has a variable fertile portion of the stromata and ascospores measuring (8.5–)10–12.5(–15) × (4.5–) 5–6.5(–7) µm, which clearly differentiate it from *X.tuberculosa*. *Xylariapolysporicola* differs from *X.tuberculosa* by having a larger apical ring (2.5–4.5 µm high × 2–3.2 µm broad) and larger ascospores ((11.5–)12.5–14.5(–15) × 5.5–8 µm) (Pan et al. 2022). Furthermore, *X.tuberculosa*, *X.petchii*, and *X.polysporicola* are clearly separated in the phylogenetic tree.

*Xylariahimalayensis* Narula & Rawla and *X.meliacearum* Læssøe also resemble *X.tuberculosa* in morphology. *Xylariahimalayensis* can be distinguished by its hairy stipe and larger ascospores ((11.5–)13–15(–15.5) × (4.5–)5–5.5(–6) µm) that lack non-cellular appendages (Ju et al. 2018). *Xylariameliacearum* can be differentiated by its stromata covered with a sulfur-yellow outer layer and larger ascospores ((19–)21.5–27.5(–31.5) × (5–)5.5–7(–8) µm) (Ju and Hsieh 2023). *Xylariatentaculata* Ravenel ex Berk. is somewhat similar to *X.tuberculosa* in morphology, but *X.tentaculata* has significantly larger ascospores (20.6–24.4 µm × 8.9–10.8 µm) (Kim et al. 2016).

##### Additional specimen examined.

China • Hainan Province, Diaoluoshan Area of Hainan Tropical Rainforest National Park; 18°43'31"N, 109°52'19"E; elevation 929 m; on fallen leaves, 26 February 2023, Xiaoyan Pan (HAFFR 63). GenBank accession numbers PQ483149 (ITS) and PQ498331 (TUB2).

## ﻿Discussion

Hainan Tropical Rainforest National Park, located in the central and southern part of Hainan Island (18°33'16"–19°14'16"N, 108°44'32"–110°04'43"E), is the region with the richest forest resources on the island, home to 4,367 species of higher plants (https://www.hntrnp.com/, accessed on 29 October 2024). Symbiosis between fungi and plants is common in nature (Thomas et al. 2016), and the rich plant diversity provides ample ecological niches for fungi, fostering many endemic species. Pan et al. (2022, 2024) described eight *Xylaria* species associated with fallen leaves and petioles. Song et al. (2022) described five new species of *Hypoxylon*, and Deng et al. (2023) published four new species of *Strobilomyces*. Based on morphological characteristics and molecular phylogenetic analysis, this paper describes two new species from Hainan Tropical Rainforest National Park, namely *X.acifer* and *X.tuberculosa*.

To date, 46 *Xylaria* species associated with fallen leaves and petioles have been discovered. Unfortunately, only 18 species’ sequences were collected in the National Center for Biotechnology Information, and this study provides sequences for two more species, leaving 26 *Xylaria* species associated with fallen leaves and petioles without genetic sequences. Phylogenetic analysis shows that the two species described in this paper are distinctly separated from other leaf-associated *Xylaria* species. Moreover, a rigorous comparison reveals that the *Xylaria* species lacking molecular sequences are also distinct from the species described in this paper, as reflected in the identification key. The eight *Xylaria* species collected by Pan et al. (2022, 2024) in Hainan grow on highly decomposed leaves. Previous research indicates that fresh leaves and decayed leaves differ significantly in nutrient content (Zeng et al. 2016), suggesting that these fungi play a crucial role in nutrient cycling within ecosystems, a view also supported by Rajtar et al. (2023). Additionally, while Ju and Hsieh (2023) summarized the *Xylaria* species associated with fallen leaves and petioles worldwide, most species’ host specificity remains unclear and requires further study. Below is the identification key for the 46 *Xylaria* species associated with fallen leaves and petioles globally (Pande 1973; Samuels and Rogerson 1990; San Martín et al. 1997, 2001; Huang et al. 2014; Ju et al. 2018; Pan et al. 2022, 2024).

### ﻿Key to the species of *Xylaria* associated with fallen leaves and petioles worldwide

**Table:** 

1	Stromata surface densely covered by yellow tomentum	***X.fulvotomentosa***
–	Stromata surface lacking yellow tomentum	**2**
2	Stromata branched, long stipes that bear one to three clavae on each terminal branch	***X.luxurians***
–	Stromata unbranched to occasionally branched	**3**
3	Fertile parts cylindrical or conical to subglobose, most perithecia gather near the top of the stromata, with several occasionally scattered below	***X.petchii***
–	Morphology of fertile parts is simpler than above, with perithecia lacking the above cluster patterns	**4**
4	Stipes glabrous	**5**
–	Stipes tomentose	**35**
5	Fertile parts filiform	**6**
–	Fertile parts not filiform	**11**
6	Ascospores with spiral germ slit	***X.meliacearum***
–	Ascospores with straight germ slit	**7**
7	Ascospores with non-cellular appendages	**8**
–	Ascospores without non-cellular appendages	**9**
8	Ascospores light brown, short fusoid	***X.fliformis***
–	Ascospores brown to dark brown, ellipsoid	***X.vagans***
9	Ascospores (15–)16.5–19(–21.5) × (5–)5.5–6.5(–7.5) µm	***X.simplicissima***
–	Ascospores length less than 13.5 µm and width less than 5 µm	**10**
10	Stromata with an acute apex, ascospores 12–13.5 × 4–5 μm	***X.eugeniae***
–	Stromata with a long acicular apex, ascospores (9–)9.5–10.5(–11) × (3.5–)4–4.5(–5) µm	***X.vermiformis***
11	Fertile parts cylindrical	**12**
–	Fertile parts not cylindrical	**27**
12	Ascospores with non-cellular appendages	**13**
–	Ascospores without non-cellular appendages	**22**
13	Stromata with an outer peeling layer split into narrow or thread-like stripes, ascospores (13.5–)14–15(–17) × (4.5–)5–6(–7) µm	***X.minuscula***
–	Stromata without an outer peeling layer or the outer peeling layer without narrow or thread-like stripes	**14**
14	Stromata with an outer peeling layer split into band-like stripes, ascospores (8–)8.5–10(–11) × (4–)4.5–5(–5.5) µm	***X.vittiformis***
–	Stromata without an outer peeling layer or the outer peeling layer without band-like stripes	**15**
15	Stromata surface lacking perithecial mounds or with inconspicuous perithecial mounds	**16**
–	Stromata surface with conspicuous perithecial mounds	**17**
16	Ascospores (22–)23.5–27(–28) × (8.5–)9–10.5(–11) µm	***X.spiculaticlavata***
–	Ascospores 8–10 × 4–6 μm	***X.kamatii***
17	Ostioles conic-papillate, tilting upwards, ascospores (14–)15–16(–17) × (6.5–)7.0–7.5(–8) µm	***X.appendiculatoides***
–	Ostioles papillate or slightly papillate	**18**
18	Stromata surface blackish brown, ascospores (15–)16.5–18(–19) × (7.5–)8–9(–9.5) µm	***X.phyllophila***
–	Stromata surface black, ascospores width almost less than 7.5 µm	**19**
19	Stromata with long, filiform apices, much longer than the fertile part	***X.diaoluoshanensis***
–	Stromata with an acute apex, shorter than the fertile part	**20**
20	Ascospores length greater than 11.5 µm and width greater than 5.5 µm	***X.polysporicola***
–	Ascospores length almost less than 11.5 µm and width less than 4.7 µm	**21**
21	Stromata surface with conspicuous to half-exposed perithecial mounds, ascospores (9.3–)10–11(–12) × 3.7–4.7 µm	***X.acifer***
–	Stromata surface with half-exposed to fully exposed perithecial mounds, ascospores 7–8(–9) × 3.3–4.3 µm	***X.tuberculosa***
22	Stromata with a striped outer peeling layer	**23**
–	Stromata without an outer peeling layer or the outer peeling layer without stripes	**25**
23	Stromata with an acuminate or mucronate apex	***X.noduliformis***
–	Stromata with a long acicular apex	**24**
24	Stromatal outer peeling layer split into narrow or thread-like stripes, ascospores (8.5–)9–11 × 4–6 μm	***X.foliicola***
–	Stromatal outer peeling layer split into band-like stripes, ascospores (10–)11–12(–12.5) × (5.5–)6–7(–7.5) µm	***X.vittatipiliformis***
25	Ascospores light brown to brown, (5.5–)6–7 × 3–3.5(–4) µm	***X.diminuta***
–	Ascospores brown to dark brown, length greater than 9 µm and width greater than 4.5 µm	**26**
26	Stromata surface dark vinaceous brown, with slight perithecial mounds	***X.phyllocharis***
–	Stromata surface blackish brown, with conspicuous perithecial mounds	***X.neblinensis***
27	Stromata surface lacking perithecial mounds or with inconspicuous perithecial mounds	**28**
–	Stromata surface with conspicuous perithecial mounds	**31**
28	Ascospores broadly ovoid to nearly globose, (11.6–)12.8–16.7(–18) × 8–15 µm	***X.clusiae***
–	Ascospores elliptical or nearly semicircular to broadly broad-elliptical, width less than 8 µm	**29**
29	Ascospores without non-cellular appendages	***X.delicatula***
–	Ascospores with non-cellular appendages	**30**
30	Stromata surface dull grayish brown, ascospores dark brown, (13–)13.5–15(–16.5) × (6–)6.5–7.5(–8	***X.hypsipoda***
–	Stromata surface dark brown to blackish, ascospores brown, 12–14 × 6–8 μm	***X.memecyli***
31	Stromata with fertile apices	***X.heloidea***
–	Stromata with acute sterile apices	**32**
32	Stromata with a long acicular apex, much longer than the fertile part	**33**
–	Stromata with an acute apex, shorter than the fertile part	**34**
33	Stromata 91–147 mm total length, ascospores (10.5–)11.5–13.5(–15) × (5–)5.5–6.5(–7.5) µm	***X.nainitalensis***
–	Stromata 3–20 mm total length, ascospores (8.5–)9.5–11(–12) × (4–)4.5–6(–6.5) µm	***X.sicula***
34	Ascospores (12–)12.5–15.5(–17) × (5–)6–7.5(–8) µm	***X.amphithele***
–	Ascospores (10–)10.5–12(–12.5) × (5–)5.5–6(–6.5) µm	***X.pisoniae***
35	Ascospores 8–9(–9.5) × 4–4.5(–6.6) µm	***X.imminuta***
–	Ascospores length greater than 10 µm	**36**
36	Ascospores with non-cellular appendages	**37**
–	Ascospores without non-cellular appendages	**40**
37	Ascospores (21.5–)22.5–24.5(–26) × (6.5–)7–8(–9) µm, with long tubular non-cellular appendages	***X.axifera***
–	Ascospores length less than 17 µm, with papillate non-cellular appendages	**38**
38	Stromata without a covering of brown tomentum, ascospores (11.5–)12.5–14(–15) × (6–)6.5–7.5(–9) µm	***X.appendiculata***
–	Stromata covered with brown tomentum	**39**
39	Ascospores (14.5–)15–16.5(–17) × (8–)8.5–9.5(–10) µm	***X.allima***
–	Ascospores (10–)10.5–12(–14) × (5–)6–7(–7.5) µm	***X.lima***
40	Fertile parts overlain by dark long spikes	***X.asperata***
–	Fertile parts without dark long spikes	**41**
41	Stromata surface with half-exposed to fully exposed perithecial mounds	**42**
–	Stromata surface lacking perithecial mounds or with inconspicuous perithecial mounds	**43**
42	Fertile parts cylindrical	***X.castilloi***
–	Fertile parts filiform	***X.duranii***
43	Fertile parts cylindrical	***X.maitlandii***
–	Fertile parts capitate	**44**
44	Stromata with rounded apices, ostioles coarsely conic-papillate	**X.aristatavar.hirsuta**
–	Stromata with acute apices, ostioles slightly papillate or papillate	**45**
45	Ascospores (10–)10.5–12.5(–14) × (5.5–)6–7(–7.5) µm	**X.aristatavar.aristata**
–	Ascospores (15–)15.5–17(–18) × (6.5–)7.5–9(–9.5) µm	***X.hispidipes***

## Supplementary Material

XML Treatment for Xylaria acifer

XML Treatment for Xylaria tuberculosa

## ﻿Additional information

### Author ORCIDs

Xiaoyan Pan https://orcid.org/0000-0002-3244-7486

Zongzhu Chen https://orcid.org/0009-0003-1464-1159

Yiqing Chen https://orcid.org/0009-0000-4416-6932

Xiaohua Chen https://orcid.org/0009-0003-5421-5509

Tingtian Wu https://orcid.org/0009-0008-8934-9255

Yuanling Li https://orcid.org/0009-0005-4826-6021

### Data availability

Datasets analyzed in this study are publicly available. All the gene sequences obtained in this article can be searched in GenBank (https://www.ncbi.nlm.nih.gov/genbank/; Table 1). All new taxa are saved in MycoBank (https://www.mycobank.org/).
